# Frequency of Central Serous Chorioretinopathy in a Tertiary Care Center in Pakistan

**DOI:** 10.7759/cureus.73249

**Published:** 2024-11-07

**Authors:** Muhammad Farhan Lodhi, Kashif Iqbal, Jawad Bin Yamin Butt, Saad Muhammad Iqbal, Irfan Akber Malik, Faisal Iqbal, Hafiz Habib Ur Rehaman Khalil

**Affiliations:** 1 Ophthalmology, Layton Rahmatulla Benevolent Trust (LRBT) Hospital Township, Lahore, PAK; 2 Ophthalmology, Akhtar Saeed Medical and Dental College, Lahore, PAK; 3 Opthalmology, Layton Rahmatulla Benevolent Trust (LRBT) Hospital Township, Lahore, PAK; 4 Gastroenterology, Lahore General Hospital, Lahore, PAK

**Keywords:** central serous chorioretinopathy, fluorescein angiography, optical coherence tomography, retinal pigment epithelium, vision disorders

## Abstract

Background: Central serous chorioretinopathy (CSC) is a significant cause of vision loss in men aged 20 to 60, characterized by serous detachment of the neurosensory retina from the retinal pigment epithelium (RPE). This study aims to assess the frequency of CSC among patients at a tertiary care center in Pakistan, offering insights into its epidemiology and management within this setting.

Methodology: A retrospective observational study was conducted at the ophthalmology department of a tertiary care center from January 2019 to December 2023. Patients aged 20 years and above diagnosed with CSC based on clinical examination, fluorescein angiography (FA), and optical coherence tomography (OCT) were included. Data were collected from electronic medical records, encompassing demographic, clinical, and treatment information. Statistical analysis was performed using IBM SPSS Statistics for Windows, version 25 (IBM Corp., Armonk, NY, USA) with descriptive statistics summarizing demographic and clinical characteristics, and chi-square tests and t-tests examining associations between variables.

Results: A total of 83 patients (112 eyes) were studied, with 83.13% being male and 16.87% female. Unilateral CSC was observed in 65.06% of cases, while 34.94% had bilateral involvement. The age group 25-40 years had the highest incidence (57.83%), with Unani medication (39.58%) and systemic hypertension (45.71%) being the most common contributing factors. The most frequent presenting symptom was vision impairment (48.19%).

Conclusion: CSC primarily affects middle-aged men, with observed associations to factors such as traditional Unani medication and systemic hypertension. This study highlights the importance of tailored treatment strategies and the need for further research into CSC's pathogenesis and management in Pakistan.

## Introduction

Central serous chorioretinopathy (CSC) is a retinal disorder that primarily affects men aged 20 to 60 years, leading to central vision loss due to serous detachment of the neurosensory retina from the retinal pigment epithelium (RPE) [1]. Characterized by a focal leakage through the RPE, CSC manifests as a well-defined area of retinal detachment, often self-resolving within three months in acute cases [2]. However, chronic CSC can develop, marked by persistent or recurrent detachments, potentially resulting in progressive RPE atrophy and permanent visual impairment [3]. The incidence of CSC and its treatment strategies have been extensively studied in various regions, yet there is limited data on its prevalence and management in Pakistan. This study aims to elucidate the incidence of CSC in a tertiary care center in Pakistan, providing insights into its epidemiology and clinical management in this region.

CSC has been recognized as the fourth most common retinal disorder in terms of prevalence [4]. While the exact pathophysiology of CSC remains elusive, it is believed to involve primary dysfunction of the RPE and choroidal vascular abnormalities [5]. Fluorescein angiography (FA) typically reveals a characteristic "smokestack" or "inkblot" pattern of dye leakage, while indocyanine green (ICG) angiography highlights choroidal hyperpermeability and delayed filling of choroidal vessels [6]. Optical coherence tomography (OCT) is a crucial diagnostic tool, providing detailed cross-sectional images of the retina, revealing subretinal fluid and morphological changes in the RPE [7-8].

Recent studies have proposed several risk factors for CSC, including systemic corticosteroid use, type A personality traits, stress, and systemic conditions like hypertension and sleep apnea [9]. Despite these associations, the precise molecular mechanisms underlying CSC are not fully understood. Emerging evidence suggests a role for the mineralocorticoid pathway in the disease's pathogenesis. Studies in animal models have demonstrated that overactivation of mineralocorticoid receptors in ocular tissues can induce phenotypes resembling acute CSC, suggesting potential therapeutic targets [10].

Therapeutic approaches to CSC vary based on the disease's acuity and chronicity. Acute CSC is often managed conservatively with observation, as spontaneous resolution is common [2]. However, in chronic or recurrent cases, interventions such as photodynamic therapy (PDT) with verteporfin, micropulse laser treatment, and mineralocorticoid receptor antagonists have shown efficacy [1,3]. PDT, particularly in half-dose or half-fluence regimens, has emerged as a preferred treatment for chronic CSC, due to its ability to selectively target abnormal choroidal vessels while preserving retinal function [1].

In Pakistan, the understanding of CSC's epidemiology and treatment is still evolving. Given the diverse population and the varying healthcare accessibility, regional data on CSC incidence and management practices are crucial for developing tailored treatment guidelines. This study aims to fill this gap by providing comprehensive epidemiological data from a tertiary care center, examining the incidence of CSC, and evaluating the effectiveness of current treatment strategies in the Pakistani context.

## Materials and methods

Study design

The retrospective observational study was conducted at the ophthalmology department of the tertiary care center, the Layton Rahmatulla Benevolent Trust (LRBT) Tertiary Teaching Eye & Hospital, Lahore, from January 2019 to December 2023. The study aimed to evaluate the incidence of CSC among patients presenting at this center. Ethical approval was obtained from the institutional review board, LRBT Tertiary Teaching Eye & Hospital, Lahore (No.2/Admn/Ex-Cer/LRBT-2024) and the study adhered to the tenets of the Declaration of Helsinki.

Patient selection

Patients were included in the study based on the following criteria.

Inclusion Criteria

Patients aged 20 years and above, diagnosed with CSC based on clinical examination and confirmed by fluorescein angiography (FA) and optical coherence tomography (OCT).

Exclusion Criteria

Patients with other retinal diseases such as age-related macular degeneration or diabetic retinopathy, significant ocular trauma or surgery, and those who had received prior treatment for CSC or had allergies to fluorescein dye.

Data collection

Data were collected from the hospital’s electronic medical records (EMR). The following information was gathered.

Demographic Data

Age, gender, occupation, and history of systemic diseases such as hypertension or corticosteroid use.

Clinical Data

Symptoms at presentation, duration of symptoms, visual acuity at diagnosis, and findings from FA and OCT.

Treatment Data

Details of treatments administered, including observation, photodynamic therapy (PDT), laser treatments, or pharmacotherapy.

Diagnostic procedures

CSC diagnosis was based on the following procedures.

Fluorescein Angiography (FA)

FA was used to identify characteristic leakage patterns such as "smokestack" or "inkblot."

Optical Coherence Tomography (OCT)

OCT was utilized to visualize subretinal fluid and morphological changes in the RPE, crucial for confirming diagnosis and monitoring disease progression.

Outcome measures

The primary outcome measure was the incidence rate of CSC. Secondary outcomes included the distribution of CSC by age and gender, the proportion of acute versus chronic CSC, and treatment outcomes.

Statistical analysis

Data analysis was conducted using IBM SPSS Statistics for Windows, version 25 (IBM Corp., Armonk, NY, USA). Descriptive statistics were used to summarize demographic and clinical characteristics.

## Results

A total of 83 patients with 112 eyes were studied. Of them, 69 (83.13%) were male patients, 14 (16.87%) were female patients; 54 (65.06%) had unilateral disease and 29 (34.94%) had bilateral disease. Out of the 54 (65.06%) patients with unilateral disease, 24 (44.44%) were right eyes and 30 (55.56%) were left eyes. The basic demographic characteristics our outlined in Table 1. 

**Table 1:** Basic demographic characteristics N (%) The data has been represented as N (%).

Variables	N (%)
Age	
25-40 years	48 (57.83%)
40-50 years	35 (42.17%)
Gender	
Male	69 (83.13%)
Female	14 (16.87%)
Laterality	
Unilateral	54 (65.06%)
Bilateral	29 (34.94%)

The most common presenting complaints were vision impairment in 40 (48.19%) patients (Table 2). 

**Table 2:** Presenting complaints N (%) The data has been represented as N (%)

Presenting Symptoms	N (%)
Micropsia	17 (20.48%)
Blue color defect	12 (14.46%)
Black spots	10 (12.05%)
Metamorphopsia	4 (4.82%)
Vision impairment	40 (48.19%)

Table 3 highlights the contributing factors for CSC categorized by age, gender, and laterality. Among patients aged 25-40 years, the most common contributing factor is the use of Unani medication 19 (39.58%), followed by allergic disease 8 (16.67%) and *Helicobacter pylori* infection 5 (10.24%). For those aged 40-50 years, systemic hypertension is the predominant factor of 16 (45.71%) patients. Males constitute 69 (83.13%) of the CSC cases, with Unani medication being the most significant contributor in 20 (28.99%) male patients. In contrast, 14 (16.87% of cases) female patients were equally affected by Unani medication and asthmatic disease 7 (50% each). Unilateral CSC is more prevalent in 54 (65.06%), with systemic hypertension in 18 (33.33%) being the leading factor, while all bilateral cases, i.e., 29 (34.94%) are associated with systemic hypertension.

**Table 3:** Contributing factors for central serous chorioretinopathy H. pylori: Heliobacter pylori, NKCM: natural killer cell-mediated The data has been represented as N (%).

Characteristics	Contributing factors										
Age		Unani medication	Asthmatic disease	Skin disease	Allergic disease	H. pylori	Systemic hypertension	Sleep apnea	Pregnancy	Renal failure	NKCM
25-40 years	48 (57.83%)	19 (39.58%)	8 (16.67%)	3 (6.25%)	8 (16.67%)	5 (10.42%)	0 (0.00%)	0 (0.00%)	5 (10.42%)	0 (0.00%)	0 (0.00%)
40-50 years	35 (42.17%)	5 (14.29%)	0 (0.00%)	0 (0.00%)	8 (22.86%)	0 (0.00%)	16 (45.71%)	0 (0.00%)	3 (8.57%)	3 (8.57%)	0 (0.00%)
Gender											
Male	69 (83.13%)	20 (28.99%)	7 (10.14%)	0 (0.00%)	2 (2.90%)	5 (7.25%)	7 (10.14%)	0 (0.00%)	0 (0.00%)	2 (2.90%)	26 (37.68%)
Female	14 (16.87%)	7 (50.00%)	7 (50.00%)	0 (0.00%)	0 (0.00%)	0 (0.00%)	0 (0.00%)	0 (0.00%)	0 (0.00%)	0 (0.00%)	0 (0.00%)
Laterality											
Unilateral	54 (65.06%)	15 (27.78%)	5 (9.26%)	0 (0.00%)	2 (3.70%)	2 (3.70%)	18 (33.33%)	4 (7.41%)	4 (7.41%)	2 (3.70%)	2 (3.70%)
Bilateral	29 (34.94%)	0 (0.00%)	0 (0.00%)	0 (0.00%)	0 (0.00%)	0 (0.00%)	29 (100.00%)	0 (0.00%)	0 (0.00%)	0 (0.00%)	0 (0.00%)

## Discussion

Central serous chorioretinopathy (CSC) is a retinal disorder predominantly affecting men aged 20 to 60 years, leading to central vision loss due to serous detachment of the neurosensory retina from the retinal pigment epithelium (RPE) [3,7]. This study aimed to elucidate the incidence of CSC in a tertiary care center in Pakistan and provide insights into its epidemiology and clinical management in the region.

The study found that CSC primarily affects middle-aged men, with a significant portion of cases involving unilateral disease. The age group 25-40 years had the highest incidence, with Unani medication and systemic hypertension being common contributing factors. The most frequent presenting symptom was vision impairment. Unilateral CSC was more prevalent, with systemic hypertension being the leading factor, while all bilateral cases were associated with systemic hypertension.

The findings of this study are consistent with previous research. Khalil et al. reported similar demographic characteristics and clinical presentations in CSC patients in Pakistan, highlighting the prevalence of unilateral disease and the common use of traditional medications [11]. Khalil et al. revealed that the male-to-female ratio was 9.1:1 and there were five angiographic patterns of CSC including inkblot, smokestack, diffuse, mixed, and non-leaking [11].

Jamil et al. also revealed a majority of males, 53 (82.8%) [12]. They identified systemic hypertension and Unani medication as significant risk factors for CSC in their cohort, aligning with the current study's observations [12]. Another study further supports these findings, noting the high incidence of CSC in middle-aged men and male gender as a possible risk factor [13].

However, some studies present differing results. Sahoo et al. found a higher prevalence of bilateral CSC in their Indian cohort, suggesting regional variations in disease manifestation [14]. Shahin's study on an Egyptian population reported a higher incidence of multifocal leaks and bilateral disease, contrasting with the primarily unilateral cases observed in the current study [15]. Laishram et al. documented a different pattern of contributing factors, with betel nut consumption being a significant risk factor in their study, which was not observed in the Pakistani cohort [16].

The existing literature and findings of this study highlight the need for tailored treatment strategies for CSC in Pakistan, considering the high prevalence of traditional medication use and systemic hypertension among patients [17,18]. Public health interventions should focus on educating patients about the potential risks associated with Unani medication and the importance of managing systemic conditions like hypertension. Further research is needed to explore the molecular mechanisms underlying CSC in this population and to develop targeted therapeutic approaches.

The study's retrospective design and reliance on electronic medical records may introduce selection bias and incomplete data. Additionally, the single-center setting limits the generalizability of the findings. However, the study's strengths lie in its comprehensive data collection and detailed analysis of demographic and clinical characteristics, providing valuable insights into the epidemiology of CSC in Pakistan.

This study provides important epidemiological data on CSC in a tertiary care setting in Pakistan, identifying middle-aged men as the primary affected group, with systemic hypertension and traditional medication use as significant contributing factors. These findings underscore the need for targeted public health interventions and further research to improve the clinical management of CSC in this region.

This study has several limitations. First, the retrospective design and reliance on medical records may introduce selection bias and incomplete data. Second, the single-center setting limits the generalizability of the findings to the broader population of Pakistan. Despite these limitations, the study's strengths lie in its comprehensive data collection and detailed analysis of demographic and clinical characteristics, providing valuable insights into the epidemiology of CSC in Pakistan.

## Conclusions

This study provides critical insights into the pattern of central serous chorioretinopathy (CSC) in Pakistan. Among the 83 patients studied, 69 (83.13%) were male, with 54 (65.06%) having unilateral disease and 29 (34.94%) having bilateral. Key contributing factors included the use of traditional Unani medication (observed in 20 (28.99%) of male patients) and systemic hypertension (notably associated with all bilateral cases). These findings highlight the need for tailored public health interventions and management strategies. Despite its limitations, this study establishes a foundation for future research to improve CSC management and patient outcomes in Pakistan.
